# Local dispersal pathways during the invasion of the cactus moth, *Cactoblastis cactorum*, within North America and the Caribbean

**DOI:** 10.1038/s41598-020-66864-3

**Published:** 2020-07-03

**Authors:** Guadalupe Andraca-Gómez, Eric Lombaert, Mariano Ordano, Rubén Pérez-Ishiwara, Karina Boege, César A. Domínguez, Juan Fornoni

**Affiliations:** 1 Departamento de Ecología Evolutiva, Instituto de Ecología, Universidad Nacional Autónoma de México, Ciudad de México, 04510 Mexico; 2 INRA, CNRS, Université Côte d’Azur, ISA, Sophia-Antipolis, France; 3 Fundación Miguel Lillo (FML), Miguel Lillo 251, T4000JFE, San Miguel de Tucumán; and Consejo Nacional de Investigaciones Científicas y Técnicas (CONICET), Instituto de Ecología Regional, Universidad Nacional de Tucumán (IER-CONICET-UNT), Tucumán, Argentina

**Keywords:** Evolution, Population genetics, Structural variation

## Abstract

*Cactoblastis cactorum*, a species of moth native to Argentina, feeds on several prickly pear cactus species (*Opuntia*) and has been successfully used as a biological control of invading *Opuntia* species in Australia, South Africa and native ruderal *Opuntia* species in some Caribbean islands. Since its introduction to the Caribbean its spread was uncontrolled, invading successfully Florida, Texas and Louisiana. Despite this long history of invasion, we are still far from understanding the factors determining the patterns of invasion of *Cactoblastis* in North America. Here, we explored three non-mutually exclusive explanations: a) a stepping stone model of colonization, b) long distance colonization due to hurricanes, and/or c) hitchhiking through previously reported commercial routes. Genetic diversity, genetic structure and the patterns of migration among populations were obtained by analyzing 10 nuclear microsatellite loci. Results revealed the presence of genetic structure among populations of *C. cactorum* in the invaded region and suggest that both marine commercial trade between the Caribbean islands and continental USA, as well as recurrent transport by hurricanes, explain the observed patterns of colonization. Provided that sanitary regulations avoiding human-mediated dispersal are enforced, hurricanes probably represent the most important agent of dispersal and future invasion to continental areas.

## Introduction

During the last decades, biological invasion studies have strongly benefited from the use of neutral molecular markers to disentangle routes of invasion^1–3^. This has helped to identify source populations, frequency of invasion events, and the geographical patterns and demographic consequences of invasive species spread^4,5^. Reconstruction of past events of invasion can help in the recognition of the mechanisms of dispersal to prevent the prevalence of invasion or further spread to additional areas^1,6–8^. However, understanding local dispersal remains a central challenge to prevent and control the economic and biodiversity costs of biological invasions.

Initially, molecular markers were used to identify sources and frequency of dispersal events of invasive species to non-native regions^1–3,9^. Whereas in some cases, these patterns explain the lower genetic variation of invasive species relative to native areas^1,10–12^, combination of migration events from different source populations have sometimes increased genetic variation within invaded regions, potentially increasing the risk of fast adaptation to novel conditions^13–16^. Once invasive species are established within non-native areas, understanding further local dispersal is a major challenge to identify potential environmental barriers and recommend control management programs^7,17^. It is possible to use highly variable molecular markers for understanding local scale dispersal and the entangled nature of the species invasion phenomena.

The cactus moth, *Cactoblastis cactorum*, is an oligophagous herbivore during its larval stage that consumes the inner tissue of cladodes of plants within the genus *Opuntia*, negatively affecting the plant survival^18^. This moth, native to South America, was used in 1924 for biological control purpose against exotic *Opuntia* species with successful results in Australia. Later, was intentionally introduced to South Africa (1933), New Caledonia (1933) and Hawaii (1950). In 1957 moth larvae, from South Africa and Australia, were introduced in the island of Nevis, seeking to control the exponential growth of native *Opuntia* populations caused by deforestation and cattle ranging^19,20^. According to a recent study, this has been the single introduction event of *C. cactorum* to the Caribbean area^21^. Since its introduction in Nevis and latter in Saint Kitts in 1957 and until 1987-1989, the moth was detected in other areas as far as Florida^22^, increasing the risk of extinction of native species^23^. The entire Florida peninsula is now invaded and the moth has spread through the Gulf of Mexico coasts^20^. According to the Texas Invasive Species Institute the moth has been detected in Brasoria County (Texas, US) in 2018 (around 800 km from the Northern Mexican border). Following the Atlantic coast of North America, it has dispersed up to Charleston County in South Carolina^24–26^. The rate of dispersal of the moth according to females flight distances was 3-6 km and 16-24 km in 2.5 years in South Africa and Australia respectively^27,28^. Hence the rapid spread throughout the Caribbean and North America probably involved additional factors. Previous reports suggest that spread among Hawaiian Islands probably occurred through island-hopping^29^ and tropical storms^30^. In turn, human-mediated dispersal and climatic events like hurricanes have been proposed as sources of dispersal in the Caribbean^21,30–32^. The threat for North American deserts is that they have the highest diversity of *Opuntia* cacti species, which may suffer from this oligophagous moth^18^. Furthermore, domesticated *Opuntia* in Mexico have a high cultural and economic value as a food resource^33^. Thus, given the risk of further spread to continental areas and the ecological, social and economic associated costs, the present study examined possible routes of local dispersal in the Caribbean and Florida to better understand the factors that may favor additional introductions of this invasive species to the continent.

A previous study using a mitochondrial gene (COI) reported that commercial transportation of ornamental cacti from Dominican Republic and Puerto Rico was the most likely route of invasion of *C. cactorum* to Florida^31^. However, a more recent study with the same marker and a more extended population survey suggested that hurricanes may have also contributed to the dispersal of the moth within the region^32^. Whereas commercial transportation of ornamental cacti has been controlled ever since the detection of this invasive species^18^, further introductions within the continent may occur via climatic events. Accordingly, the goal of this study was to further advance in the comprehension of the patterns of dispersal of *C. cactorum* by using nuclear microsatellites and computational Bayesian approaches. This approach will allow us to add a new piece of evidence to determine whether dispersal through trade, climatic events (hurricanes) or both, better account for the current genetic structure of *C. cactorum* in North America.

## Material and Methods

### Sample collection

**Figure 1:**
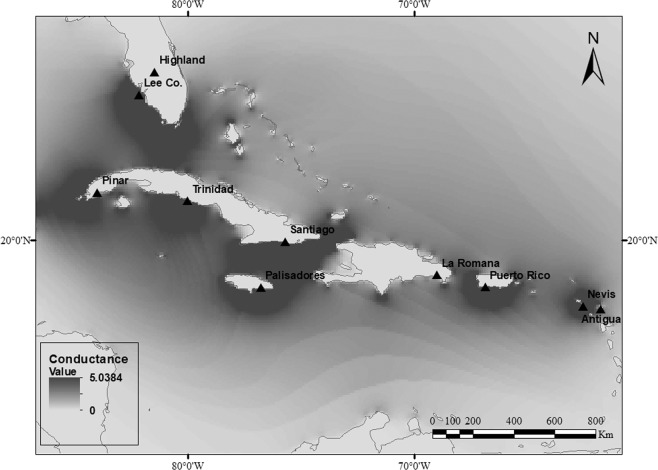
Geographic location of the invading Caribbean populations of *Cactoblastis cactorum*. The location is on the conductance map for the Caribbean and Atlantic sea obtained with the CIRCUITSCAPE program based on hurricane incidence values, the darkest areas show those with the highest probability of flow. This conductance map was built using the isopletes shown at Lugo *et al*.^48^ and available in NOAA (https://www.nhc.noaa.gov/).

From 2011 to 2012, a total of 229 larvae of the cactus moth, *C. cactorum*, were collected from 10 invasive sites within the Caribbean region and Florida (USA) (N = 185) (Fig. 1), and two native ones in Argentina (N = 44) from where the moth was initially collected and transported for biological control of ruderal *Opuntia* species in Australia in 1924 (Table 1). Within each collecting site, only one larva was selected per individual host of *Opuntia* spp. to avoid over-representation of genotypes belonging to the same genetic family. All samples were stored in 95% ethanol until DNA extraction.

**Table 1:** Description of studied invasive and native populations of *Cactoblastis cactorum* sampled in the Caribbean, Florida and in the native region. Average number of alleles (NA), observed heterozygosity (Ho), expected heterozygosity (He), allelic richness (RA), and Fixation Index (*F*_IS_) are provided. Standard deviations are indicated in parenthesis. *significant *P* < 0.05 *F*_IS_ values. Statistics calculated with 10 microsatellites.

Population	Country	Coordinates	Sample size	Registration date	NA	Ho	He	RA	*F*_IS_
INTRODUCED									
1. Antigua	Antigua	16.998 N 61.75459 W	15	1958	3 (1.33)	0.314 (0.175)	0.425 (0.213)	1.849 (0.439)	0.266*
2. Nevis	Nevis	17.11423 N 62.54811 W	6	1957	3 (1.41)	0.371 (0.225)	0.479 (0.222)	1.992 (0.476)	0.239*
3. Guanica	Puerto Rico	17.96472 N 66.84639 W	24	1963	2 (0.94)	0.282 (0.27)	0.258 (0.252)	1.494 (0.475)	-0.14
4. La Romana	Dominican Republic	18.4959 N 68.98981 W	20	Unknown	3.3 (1.88)	0.393 (0.183)	0.47 (0.226)	1.972 (0.537)	0.14
5. Palisadores	Jamaica	17.942317 N 76.762844 W	14	Unknown	2.2 (1.22)	0.193 (0.205)	0.23 (0.248)	1.453 (0.469)	0.167
6. Santiago de Cuba	Cuba	19.96175 N 75.68988 W	29	1980	2.7 (0.82)	0.309 (0.178)	0.35 (0.197)	1.687 (0.378)	0.109*
7. Trinidad	Cuba	21.76201 N 80.00963 W	30	Unknown	3.1 (2.33)	0.38 (0.29)	0.369 (0.28)	1.765 (0.618)	-0.027
8. Pinar del Rio	Cuba	22.13986 N 83.97028 W	30	Unknown	2.7 (0.82)	0.35 (0.199)	0.37 (0.226)	1.741 (0.444)	0.034
9. Highlands	Florida (USA)	27.46667 N 81.447 W	15	1990	2.3 (1.16)	0.46 (0.296)	0.386 (0.256)	1.767 (0.546)	-0.199
10. Lee Co.	Florida (USA)	26.451417 N 82.1232 W	2	Unknown	1.6 (0.69)	0.25 (0.264)	0.283 (0.315)	1.600 (0.663)	0.167
NATIVE									
11. Ayuí	Argentina	31.19545 S 58.04662 W	24	—	3.3 (1.49)	0.383 (0.277)	0.487 (0.214)	2.016 (0.463)	0.213*
12. Yuquerí	Argentina	31.38195 S 58.128863 W	20	—	3.8 (1.31)	0.505 (0.263)	0.537 (0.247)	2.185 (0.560)	0.062

### DNA extraction and microsatellite analyses

We performed total DNA extraction with DNEasy blood and tissue kits (Qiagen, MD, USA) following the manufacturer protocol. Population genetic variation was determined using nuclear microsatellites developed by Genetic Marker Services (http://www.geneticmarkerservices.com). We removed loci that did not amplify among the 29 primers pairs tested. We chose 14 potential polymorphic loci (Table 2) and labeled with fluorophores (Applied Biosystems) for fragment analysis. Each multiplex PCR mixture (5 µL) contained 2.5 µL RadyTaq (Qiagen cat. 206143) and 1 µL DNA template (20 ng) 5pmol for each fluorescent labeled primers. PCR were performed through touchdown reaction starting with initial heat activation at 95 °C for 10 min followed by 6 denaturation cycles of 94 °C for 1 min, annealing for 1 min and 1 min of extension at 72 °C; annealing cycle temperature began at 60 °C or 57 °C and decreased 1 °C every cycle. The PCR reaction ended with two stages of 12 cycles each (57° and 56°, respectively) and final elongation of 72 °C for 5 min. The PCR product was diluted and run on an ABI 3730xl automated capillary sequencer. The allele size was manually scored using a Liz 600 size standard (Applied Biosystems) in GeneMarker (V2.2.0) (Soft Genetics LLC, State College, Pennsylvania, USA).

**Table 2:** Fourteen nuclear microsatellites amplified on *Cactoblastis cactorum* (subscripts correspond to the groups for multiplex PCR reactions).

Id	GenBankID	Repeat Motifs	Primer sequence (5′-3′)	size range
cc11^2^	MN659347, MN659348	(AC)9	F:CCGGTCGTAACTGGCTTAAA R:TCATCCTTTTTGTCCCACTCT	192–234
cc12^1^	MN659349, MN659350	(TC)15	F:CACAATGGCTCCCGACTACT R:ACTGGCTGGTCTGTCTGGTT	222–286
cc13^1^	MN659351, MN659352	(GT)9	F:CCATCATTTGGGGGAAAAA R:ATGGTGACACTGGCAGAATG	115–149
cc15^1^	MN659353, MN659354	(GT)8	F:CGAGCAGGCTCATACCACTT R:CATGACGTTCTCGGATTATGG	95–119
cc16^3^	MN659355, MN659356	(GA)12	F:GCGGGAAGCTCATTGTTTAT R:CGGTCTTTCTTTTTGCATCA	152–190
cc3^4^	MN659357, MN659358	(GT)10	F:TAAACATAAACACAGTGCTGCC R:TGAGGTTCCAAATTAATGGTCAG	138–164
cc4b^4^	MN659359, MN659360	(GT)8	F:TGTGTGCGTGTTATTGCGTA R:GAGTTGCATGTTAGTCGCATTT	80–130
cc59^2^	MN659361, MN659362	(GT)11	F:CAACTTCTCTGCTCTCGTTC R:CGACATTAACTTCGATCAAC	101–117
cc6^1^	MN659363, MN659364	(AC)11	F:CCCTTGATGATCACCTTTCG R:TTTAACCCTCCACGCAAAAC	119–143
cc60^3^	MN659365	(TG)11	F:AGGTCAATGTGTGTGTGTGT R:GTACCTCTATCAAGAGTTTCG	85–117
cc63^2^	MN659366	(AC)8	F:CACCAGCCAAGGTCAGTCTT R:CAAACGTCGTCATTAACATGG	130–136
cc65^3^	MN659367, MN659368	(AC)10	F:TTCCTGTTTCAAGCCCTTTC R:AATCGTGGGATTTGCCATTA	176–220
cc6b^2^	MN659369	(AC)9	F:CACACGAGATAATGTGATAACAGG R:AATGTGTGTGTGTGCGTGTG	81–122
cc7b^4^	MN659370, MN659371	(GT)11	F:CATAAGTATCCGGGACATGC R:TTTCCTACATAAAAACATTTCAACCA	130–162

### Basic population estimators of genetic variation

Genetic variation within sampled populations was characterized using the mean number of alleles per locus (NA), the allelic richness (AR), the mean expected- (He)^34^ and observed heterozygositiy (Ho), the Fixation Index (*F*_is_) and estimation of *F*_ST_^35^ between each pair of sampling site using FSTAT 2.9.3.^36^. Mean allelic richness (AR) was calculated using the rarefaction method of Leberg^37^. Exact test for deviation from Hardy-Weinberg equilibrium were performed with GENEPOP^38^. We used FreeNA to determine the frequency of null alleles using EM algorithm^39^. Pairwise population differentiation was tested using only those loci that were in H-W equilibrium in more than 50% of the populations.

Genetic clustering of populations was examined using STRUCTURE^40^ (v 2.3.3). We chose the admixture model with correlated allele frequencies. Two analyses were performed, one including all native and invaded population samples, and one including only populations from the invaded area. The first analysis examined whether genetic differentiation has occurred after the human-mediated journey of *C. cactorum* from its native Argentina to the Caribbean (1957). The second analysis was performed to explore genetic structuring within the invaded Caribbean only. Because groups of larvae were collected from discrete sites, sampling locations were used as prior information^41^. Each run consisted of a burn-in period of 10^5^ Markov Chain Monte Carlo (MCMC) iterations, followed by 10^6^ iterations. 20 replicated runs were carried out for each value of the potential number of clusters (*K*) set, between 1 and 9. STRUCTURE HARVESTER^42^ (available at http://taylor0.biology.ucla.edu/structureHarvester/) was used to collating output results from STRUCTURE and to determine the uppermost level of structure using the method of Evanno *et al*.^43^. Because STRUCTURE assumes the absence of null alleles and H-W equilibrium for all loci, analyses were performed using 10 out of 14 microsatellites that fulfilled the required assumptions.

### Isolation genetic and connectivity in the invaded region

The hypothesis that genetic differentiation between a pair of populations is a linear function of the geographic distance between them was examined through a Mantel test that correlates genetic and geographic distance matrices. Paired genetic distances were estimated as [*F*_ST_/ (1-*F*_ST_)] and geographic distances were estimated as the logarithm of the Euclidean distances (b_log_) using GenAlEx^44^ (v.6.4). Isolation was also tested considering that hurricanes may also shaped the genetic structure of populations in this area. To explore the relationship between genetic distances and the frequency of hurricanes incidence across pairs of populations, a resistance matrix was constructed using the CIRCUITSCAPE program^45^ (Fig. 1). Based on a previous subdivision of the geographic area using a grid with the probability of incidence of hurricanes (categories 1 to 5) following NOAA^46–48^. Pairs of populations connected by cells with high incidence of hurricanes were assigned a high conductance value (i.e., low resistance to migration). This rationale was applied to all pairs of populations to construct a resistance matrix (indicated by light areas in Fig. 1). To correlate the resistance matrix against the genetic distance matrix controlling for the geographic distance among populations, a partial Mantel test with 10,000 permutations was implemented.

### Approximate Bayesian Computational analysis

**Figure 2:**
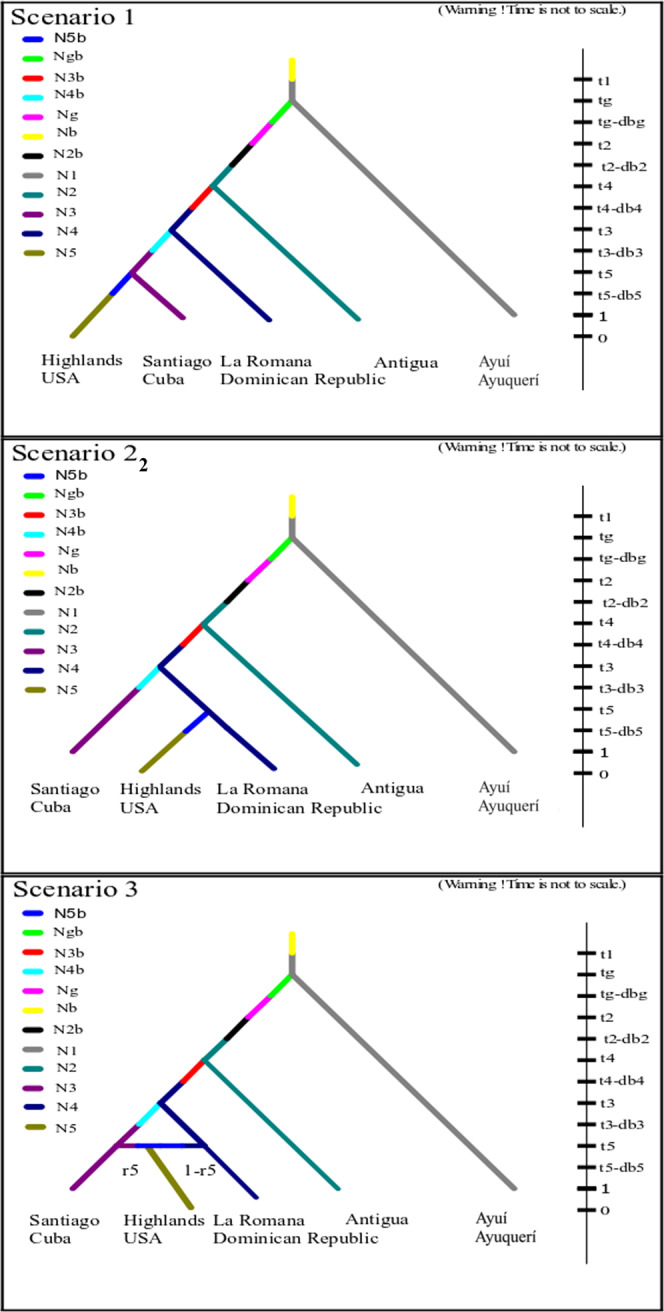
Scheme of the three competing scenarios evaluated with the ABC model. The prior distribution values of parameters are described in Table 3 (0 = year 2012, assuming two generations per year back in time). The scenarios was built and evaluated with DIYABC (V. 2). The edition was made in the Inskape program.

To provide a quantitative evaluation of the dispersal routes of the cactus moth into Florida (USA), an Approximate Bayesian Computational Analysis was performed with DIYABC^49^. Analyses were performed with ten microsatellites that were at Hardy-Weinberg equilibrium. This approach uses genetic information provided by microsatellites data and historical data given by dates of first observation within invaded regions. Three hypothetical invasion scenarios were examined (Fig. 2), and parameter values were drawn from prior distributions (Table 3). In all three cases we used the native Argentinian population of Ayuí as the initial source of invasion. This population was genetically close in average with the invasive populations (Paired *F*_ST_ = 0.225). Since moths were taken from Argentina to Australia, and then to South Africa, the analysis considered a “ghost” population representing a non-sampled population that may represent this step. Given the location and date of introduction to the Caribbean, the sampled population of Antigua was considered the source population that initiated the invasion in the region. Antigua was preferred over Nevis because of the larger number of individual larvae in our collection, the high genetic similarity between both islands (Paired *F*_ST_ = 0.012, N.S.) and the short difference in time of introduction between them (1 year). The following step in the invasion process was characterized by the population La Romana (Dominican Republic), given that the moth was detected in the island of Desecheo, located between Puerto Rico and Dominican Republic in 1963. After this step, hypotheses differ in how the cactus moth reached Florida. In the first hypothesis, Cuba, represented by the sampled population of Santiago, is the source of the Floridian invasive population (Fig. 2, Scenario 1). Due to US embargo to Cuba, any dispersal of the moth from Cuba to Florida is more likely related to environmental agents (such as hurricanes) rather than commercial transportation. The second hypothesis considers that moths belonging to La Romana (Dominican Republic) directly invaded Florida (Highlands) (Fig. 2, Scenario 2). This route constitutes the recorded trajectory of commercial traffic to Florida of ornamental cacti infested with *C. cactorum* from Dominican Republic and Puerto Rico detected in 1989^50^. However, we cannot rule out the possibility that hurricanes also contributed to dispersal among these areas. The third hypothesis considers that the population of Highlands (Florida) was the result of the admixture of genotypes belonging to Cuba and Dominican Republic (Fig. 2, Scenario3).

**Table 3:** Parameters used for data simulation in the three competing scenarios using ABC analyses.

Model parameter	Minimum	Maximum	Distribution shape
Population size (number of diploid individuals)			
N_i_	10	3000	Uniform
Bottleneck generation number			
db_i_	1	10	Uniform
Effective number of founders			
n_i_b	2	200	Log-Uniform
Number of generations since introduction			
T_1_ (Argentina)	200	1000	Uniform
T_g_ (unsampled population)	172	172	
T_2_ (Antigua)	104	104	
T_3_ (Dominican Republic)	80	100	Uniform
T_4_ (Cuba)	64	74	Uniform
T_5_ (USA)	44	54	Uniform
Admixture rate (Model 3)			
r5	0.1	0.9	Uniform

Given that *C. cactorum* usually has two generations per year^27,28,51^, this generation time was used to scale coalescent time in all scenarios. Dates of introduction to Australia (Tg) and Antigua (T2) were given as fixed values based on historical data of the intentional introduction events^18^. In the other areas (T_1_ (Argentina), T_3-5_ (Dominican Republic, Cuba and USA); Table 3), a minimum and maximum value was assigned to each time parameter, with the dates of first observation of the insect as the lower boundary (Table 3). We simulated three million microsatellite data sets (one million for each scenario). Subsequently, the probability for each scenario was inferred by polychotomous logistic regression on the 1% of the simulated data sets closest to the observed data set^49^. The selected scenario was chosen as that with the highest posterior probability value. To evaluate the robustness of the analysis, we used pseudo-observed simulated data sets to quantify the type I error rate (risk to exclude the focal scenario when it is the true one) and the type II error rate (risk to select the focal scenario when it is false)^49^. All simulations and ABC analyses were carried out in DIYABC (v.2) sofware^52^.

## Results

No evidence of linkage disequilibrium was detected for all 14 microsatellite loci. Four loci were excluded from the analysis because they had more than 20% of null alleles and were not at Hardy-Weinberg equilibrium (i.e., cc7b, cc16, cc65, cc6b). With the remaining ten microsatellites loci we found that the native population from Yuquerí (Argentina) had on average the highest number of alleles (3.8), while the lowest average number of alleles (1.6) was observed in one of the most recent invasive population (Lee Co., USA) (Table 1). Allelic richness (AR) was higher in native range (2.016–2.185) than invaded populations (1.453–1.992; Table 1). A deficit of heterozygotes was detected in one of the native populations (Ayuí), and in three of the Caribbean invaded populations (Antigua, Nevis and Santiago; Table 1). On average, lower levels of heterozygosity were recorded for invaded than native populations (Table 1).

Pairwise comparisons indicated significant differentiation between the native Argentinian populations and all invaded populations in the Caribbean and Florida (mean *F*_ST_ = 0.229, range = 0.128–0.49). Within the invaded region, genetic differentiation ranged from almost no differences to rather high levels of genetic differentiation (Table 4). The average level of differentiation within the invaded region was *F*_ST_ = 0.185. In general, early invaded populations (Nevis and Antigua) presented lower levels of differentiation with the rest of the populations in the region than recently invaded populations. The Jamaican population (Palisadores) presented the greatest level of genetic differentiation within the Caribbean (mean *F*_ST_ = 0.310; Table 4), whereas Florida populations had a low level of differentiation with population La Romana (Dominican Republic) and low to moderate differentiation with two of the closest Cuban populations (Pinar del Río and Trinidad; Table 4).

**Table 4:** Pairwise F_ST_ of *Cactoblastis cactorum* populations. Values in bold were not statistically significant. The adjust for multiple comparisons was α = 0.0006.

	Antigua	Nevis	Puerto Rico	La Romana	Palisadores	Santiago	Trinidad	Pinar del Rio	Highlands	Lee Co.	Ayuí
Nevis	**0.012**										
Puerto Rico	0.204	0.164									
La Romana	0.038	**0.019**	0.131								
Palisadores	0.304	0.337	0.470	0.284							
Santiago	0.095	0.056	0.269	0.112	0.257						
Trinidad	**0.099**	0.057	0.206	0.070	0.324	0.137					
Pinar del Rio	0.206	0.156	0.394	0.194	0.302	0.148	0.127				
Highlands	0.166	0.122	0.265	0.101	0.397	0.193	0.127	0.232			
Lee Co.	**0.114**	**0.092**	**0.436**	**0.097**	**0.426**	**0.110**	**0.095**	**0.076**	**0.100**		
Ayuí	0.160	0.142	0.185	0.190	0.430	0.296	0.251	0.302	0.154	0.137	
Yuqueri	0.155	0.128	0.206	0.232	0.390	0.290	0.271	0.296	0.208	0.156	0.100

**Figure 3:**
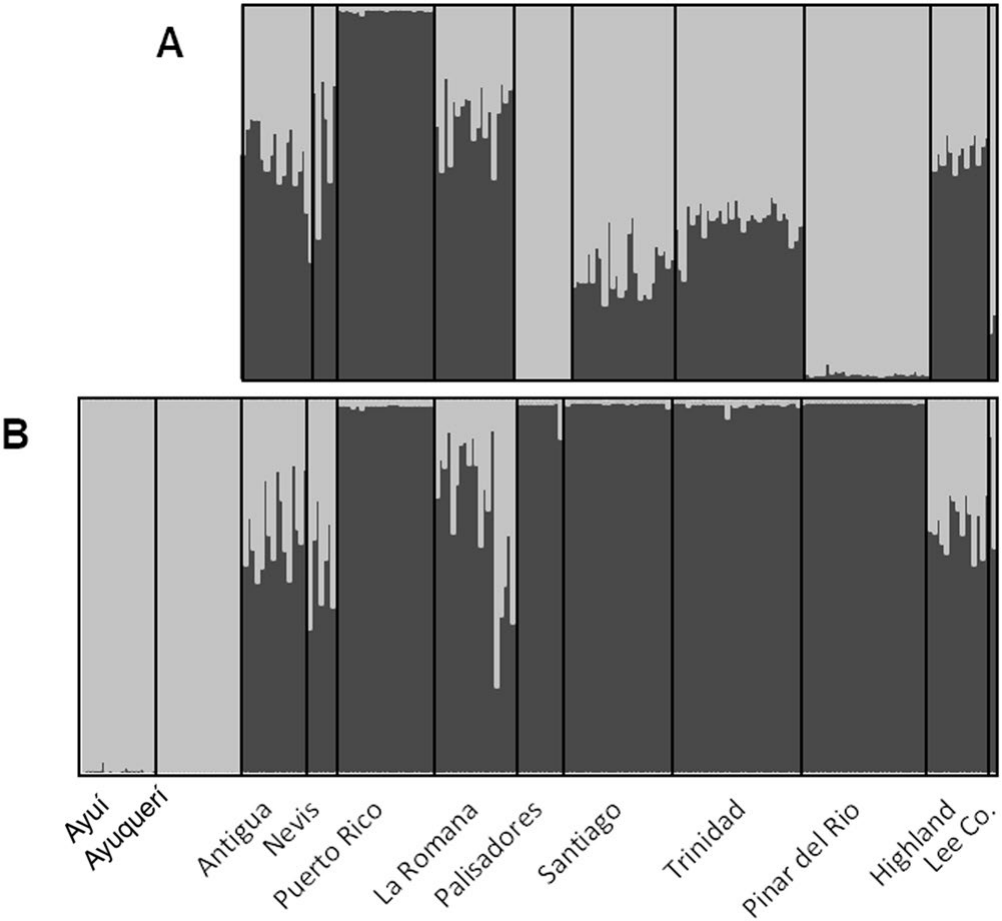
Ancestry estimation based on the Bayesian clustering method STRUCTURE in the *Cactoblastis cactorum* samples. (**A**) Genetic clustering of the 10 introduced populations, assuming two population clusters (*K* = 2). (**B**) Genetic clustering of the 10 introduced populations and the 2 native populations, assuming two population clusters (*K* = 2). Note: each vertical line corresponds to an individual and the shades of gray represent the probability of belonging to a group. Individuals are grouped by population sample.

Clustering genetic analyses using STRUCTURE on the whole dataset revealed the presence of two main groups (*K* = 2, with the delta *K* method) distinguishing native from all invasive populations (Fig. 3B), and tend to confirm a single introduction event. When analyzing only the group of invasive populations, *K* = 2 is also selected (Fig. 3A). While individuals from Puerto Rico, Palisadores and Pinar del Rio belong to a well-defined cluster, the rest of the populations showed an admixed pattern. Analyses of isolation by distance and connectivity including only the invading populations indicated a low but almost significant level of isolation by distance (r = 0.165; *P* = 0.05). On the other hand, the isolation by resistance hypothesis based on hurricane and wind currents was significantly supported (r = 0.435; *P* = 0.008).

Results from Approximate Bayesian Analyses (ABC) support quiet well the hypothesis described in the third scenario (Fig. 2), due to (1) a higher consistency between simulated and sampled data (i.e. the highest posterior probability; *P* = 0.7798), (2) non-overlapping confidence intervals, and (3) low Type I and mean Type II errors (0.038 and 0.000; respectively). This dispersal scenario considers that the invasion to Florida (represented by the Highlands population) was funded by insects belonging to at least two sources of dispersal, one from Dominican Republic and the other from Cuba (represented in the analysis by the Santiago population; Fig. 2). The posterior probability estimation of parameters of model three assigned a median admixture rate of *R* = 0.4.

## Discussion

During the 1981–1991 decade, North American customs authorities intercepted *Opuntia* plants infested with *C. cactorum*. Seizures occurred in plant shipments sent from the Dominican Republic to Miami and in luggage to Dallas Texas International Airport^53^. Commercial trade of ornamental cacti from the Dominican Republic to the United States of America coupled with the constant influx of American tourists to the Caribbean islands lead to the proposal that human-mediated dispersal has been the major agent of migration to the continent. Similarly, the comparison of the mitochondrial haplotypes from eastern populations of *C. cactorum* of Florida and those of Dominican Republic supports this idea^21,31,32^. Despite other potential mechanisms of dispersal as island-hopping and tropical storms or hurricanes^30^, the present study support the genetic similarity and likelihood of migration between La Romana (Dominican Republic) and Highlands (Florida) populations. However, the distribution of genetic variation and ABC analyses of invasion routes within the Caribbean region also suggest the presence of other nonexclusive routes of dispersal.

In the Caribbean and North Atlantic region, hurricanes are a ubiquitous temporary phenomenon with consistent wind current patterns. Studies of genetic variation have shown that hurricanes and marine currents influence the patterns of dispersal and constitute a source of variation in ecological and demographic processes^54^. Wind has been identified as one of the most important factors promoting the spread and long-distance dispersal of invasive insect species^7,55^ and to enhance distance and/or speed of dispersion as in the case of the monarch butterfly^56^, the cattle egret^57^ or the mealy bug, *Oracella aculata*^58^. Also, the grasshopper *Eumetopina flavipes*, vector of the sugarcane virus Ramu, entered Australia from New Zealand favored by wind currents^59,60^. Wind is also responsible for long-distance dispersal of the wasp *Megastigmus schimitscheki* in France^7^. Although previously suggested, the role of hurricanes on local dispersal of *C. cactorum* within the Caribbean was not examined until recently^32^. Our previous results using mitochondrial COI^32^ coupled with the findings of this study using nuclear microsatellites, are consistent and provide new pieces of evidence supporting the role of hurricanes on the spread of *C. cactorum* toward mainland North America. In comparison with the isolation by distance model, the isolation by resistance migration approach was a much better predictor of the current pattern of genetic variation of *C. cactorum* in the Caribbean and Florida. This finding suggests that hurricanes were one of the main drivers of dispersal from the Caribbean to mainland North America as previously shown using a more conserved mitochondrial marker^32^. In addition, ABC analyses of the invasion routes supported our hypothesis that moths entered Florida both through commercial transportation from the Dominican Republic and from Cuba as a consequence of climatic events (hurricanes); whereas trade with Cuba was suspended as a result of the embargo. Moreover, this finding suggests that hurricanes may have also dispersed *C. cactorum* from its initial introduction in Nevis, Antigua and Montserrat to other islands onto the Caribbean region. Historical records of hurricanes in the region (https://www.nhc.noaa.gov/) further support this statement. After the initial human-mediated introduction of *C. cactorum*, three hurricanes connected the Lesser Antilles and the Dominican Republic between 1963 and 1967, four impacted the south of Cuba and the Dominican Republic between 1975 and 1980, and two passed from Cuba and impacted the USA in 1985 and 1987. Nevertheless, the presence of the moth in the lower Bahamas in 1983^61^, and the possibility of island hopping, as recorded in the Hawaiian archipelago, can also represent a possible route of dispersal towards Florida. During the last decades at least three hurricanes impacted the Bahamas before reaching the Atlantic coast of Florida (Mitch in 1988; Andrew in 1992, and Katrina in 2005). Hence a more extended survey of populations in this area of the Caribbean will provide further insight on the dispersion routes across the region.

The process of invasion is generally associated with the loss of genetic variation due to successive demographic bottlenecks as the invasion of new locations proceeds^62^. This process can be counteracted if repeated invasion events occur in a given location, thus increasing the amount of genetic variation to equivalent, or larger levels of variation, than that observed on the native region^17,63^. Our analyses revealed that populations of *C. cactorum* from the Caribbean and Florida, sustained less genetic variation than in the native region, likely as a consequence of genetic drift. Given that present populations of *C. cactorum* outside the Caribbean region are located more that 8,000 km away in other biogeographical areas and continents, the genetic structure results support previous analyses using mitochondrial markers suggesting that the insect entered the Caribbean probably once in 1957^21^. Similar to the Lepidopteran tomato pest, *Tuta absoluta*, in Africa and the Mediterranean Basin^62^, the occurrence of just one introduction of the cactus moth was enough to allow its spread throughout the Caribbean despite the lower genetic variability than the source population from South America. Unlike the tomato pest and other invasive insect species^1,9^ significant genetic differentiation was detected in the invaded region suggesting again a possible effect of drift. A more complete survey of invaded populations in Australia, South Africa and in other continents will help to decipher whether the magnitude of reduction in genetic variation will affect the adaptive potential of the moth into other continental areas with different climatic conditions as those of the Caribbean and Florida.

The combined effect of hurricanes and genetic drift reducing genetic variation through space will likely affect the evolutionary potential of the moth during future introductions to the continent. Hence, monitoring programs of climatic events like hurricanes in the region and environmental genomics surveys will add new insight to better understand possible barriers to the expansion of the moth into continental areas.
